# Dysregulation of Transposon Transcription Profiles in Cancer Cells Resembles That of Embryonic Stem Cells

**DOI:** 10.3390/cimb46080505

**Published:** 2024-08-05

**Authors:** Anna I. Solovyeva, Roman V. Afanasev, Marina A. Popova, Natella I. Enukashvily

**Affiliations:** 1 Lab of the Non-Coding DNA Studies, Institute of Cytology, Russian Academy of Sciences, 194064 St. Petersburg, Russia; anna.solovyeva@zin.ru (A.I.S.);; 2 Zoological Institute of Russian Academy of Sciences, 199034 St. Petersburg, Russia; 3 Applied Genomics Laboratory, SCAMT Institute, ITMO University, 191002 St. Petersburg, Russia; 4 Department of Cytology and Histology, St. Petersburg State University, 199034 St. Petersburg, Russia

**Keywords:** transposons, retroviruses, embryonic stem cells, pluripotency, repeatome, tumor, HERVH, HERV-Fc1, HERV-Fc2, cancer cell lines, tumor microenvironment, cancer-associated fibroblasts

## Abstract

Transposable elements (TEs) comprise a substantial portion of the mammalian genome, with potential implications for both embryonic development and cancer. This study aimed to characterize the expression profiles of TEs in embryonic stem cells (ESCs), cancer cell lines, tumor tissues, and the tumor microenvironment (TME). We observed similarities in TE expression profiles between cancer cells and ESCs, suggesting potential parallels in regulatory mechanisms. Notably, four TE RNAs (HERVH, LTR7, HERV-Fc1, HERV-Fc2) exhibited significant downregulation across cancer cell lines and tumor tissues compared to ESCs, highlighting potential roles in pluripotency regulation. The strong up-regulation of the latter two TEs (HERV-Fc1, HERV-Fc2) in ESCs has not been previously demonstrated and may be a first indication of their role in the regulation of pluripotency. Conversely, tandemly repeated sequences (MSR1, CER, ALR) showed up-regulation in cancer contexts. Moreover, a difference in TE expression was observed between the TME and the tumor bulk transcriptome, with distinct dysregulated TE profiles. Some TME-specific TEs were absent in normal tissues, predominantly belonging to LTR and L1 retrotransposon families. These findings not only shed light on the regulatory roles of TEs in both embryonic development and cancer but also suggest novel targets for anti-cancer therapy. Understanding the interplay between cancer cells and the TME at the TE level may pave the way for further research into therapeutic interventions.

## 1. Introduction

One of the most striking results of the Human Genome Project was the discovery that our genome is non-coding. Exons of coding genes comprise only 2% of human DNA. More than 90% of a mammalian genome is composed of non-coding sequences, with approximately 55% of these sequences being repetitive [1]. They are classified into two large groups: “tandem repeats” and “dispersed repeats.” Tandemly repeated DNA is organized as multiple copies of homologous DNA monomers arranged in a head-to-tail manner. Dispersed repeats, also known as transposable elements (TEs), selfish genetic elements, jumping genes, or parasitic DNA, are dispersed repetitive sequences, with their single copies scattered throughout the genome. They can move within a genome by a mechanism called transposition [2]. TEs are the largest component of the human genome, accounting for 45% of its content [3]. They are divided into two classes, retroelements (class I) and DNA transposons (class II), based on their transposition mode and sequence organization [4]. Retroelements, due to their replicative transposition and ongoing activity, are the major type of TEs in the human genome. There are different types of retrotransposons, including endogenous retroviruses (ERVs), which are characterized by the presence of long terminal repeats (LTRs) and non-LTR retrotransposons. Non-LTR retrotransposons are classified into three types: long interspersed nuclear elements (LINEs), short-interspersed nuclear elements (SINEs), and SVA elements (SINE, VNTR, and Alu. composite elements). Non-LTR retrotransposons are characterized by polyA-tail and target site duplications. LINEs make the largest contribution to the human genome at 20.4%, followed by SINEs (13.1%), LTRs (9.1%), and SVAs (0.1%) [5].

TEs are considered both as ‘parasites’ and ‘symbionts’ of a eukaryotic genome [6]. On one hand, they can induce insertional mutations that can accelerate the development of cancer, neurodegenerative disorders, and schizophrenia. On the other, they can be neutral or even serve as a tool for rapid human evolution [7,8,9]. The role of TEs in evolution as a driver of clonal diversity has been previously demonstrated [10]. TEs are also involved in chromatin 3D organization and telomere maintenance and are a source of new regulatory elements [6]. Usually, TEs are silenced through several mechanisms, but they may be reactivated during periods of high cellular plasticity, such as development, regeneration, and aging [2,6,11,12]. Some ERVs may assist their host genomes in the innate immune response to viral infections [9]. One of the most striking examples of TEs as eukaryotic cell symbionts is their role in pluripotency maintenance.

During normal development, the transcriptional regulation of TEs is tightly controlled by the cell. Although some TEs are highly expressed in embryonic stem cells (ESCs), they are silenced in terminally differentiated cells [13,14]. In contrast, during tumorigenesis and cancer transformation, some TEs lose silencing and their transcription resumes [15]. The LINE-1 sequence contains an RNA-polII promoter, which is hypomethylated in many malignancies. In this hypomethylated state, the promoter is reactivated, enabling retrotransposition and increasing genetic instability. This is associated with poor prognosis in lung and colon cancers [16]. ERVs can also provide a promoter for long non-coding RNA (lnсRNA) transcription in some cancers [17,18]. ERV transcripts are involved in the formation of dsRNA with cellular RNA. These transcripts can silence some anti-tumor genes or stimulate a cellular antiviral response [16].

It has been hypothesized that cancers may reawaken developmental TEs to drive embryonic hallmarks for cancer progression [19,20]. Comparative studies on TEs expression in ESCs and cancer cells may provide insight into new targets for anticancer therapy. The data on TEs activation in cancer have been obtained from different sources, including cancer cell lines, cancer tissues, and primary cell cultures [13,21]. In studies analyzing transcriptomes from cancer tissues, the role of the microenvironment is often underestimated, owing to the fact that libraries for RNA sequencing are frequently created from bulk tumor tissues. However, the examination of bulk tumor material or of only cancer cells in culture could mask the contribution of tumor stromal cells that make up the majority of the tumor mass. To distinguish cancer cells from the tumor microenvironment (TME), single-cell sequencing is necessary.

The importance of the TME in the development and advancement of primary cancer is widely acknowledged. Tumors grow in a complex and dynamic stroma composed of stromal cells, immune cells, matrix proteins, and soluble factors. This microenvironment provides stimuli for tumor survival, growth, and invasiveness [22,23]. Currently, there is a special focus on cell–cell communication in the tumor microenvironment mediated by non-coding RNA [24,25]. LncRNA interacts with other cellular macromolecules such as DNA, protein, and RNA and is involved in tumor/stroma crosstalk, which stimulates a permissive TME [24,26]. Cancer cells and immune and stromal TME cells have distinct roles in cancer progression. Therefore, they should have different TE transcription profiles.

The objective of this study was to evaluate the expression of TEs in ESCs, tumor tissues, and corresponding cancer cell lines, as well as in the tumor microenvironment (TME) cells. Lung adenocarcinoma (LUAD) and neuroblastoma (NB) were chosen as two of the most common and aggressive solid tumors, while multiple myeloma (MM) was chosen as an example of a hematological malignancy. The data presented may offer insight for future functional studies and identify potential targets for further investigation of the cross-talk between cancer cells and the tumor microenvironment. The data on TE specific for cancer, but not ESCs, can be used for drug design.

## 2. Materials and Methods

### 2.1. Bulk RNA-Seq Data Processing

RNA-seq data of ESCs and tumors available in SRA (sequence read archive, https://www.ncbi.nlm.nih.gov/sra, last accessed on 15 April 2024) were used in the study. A list of the transcriptomes used is provided in Table 1. Fibroblast sequencing data were used as an example of normal cells.

**Figure 1:**
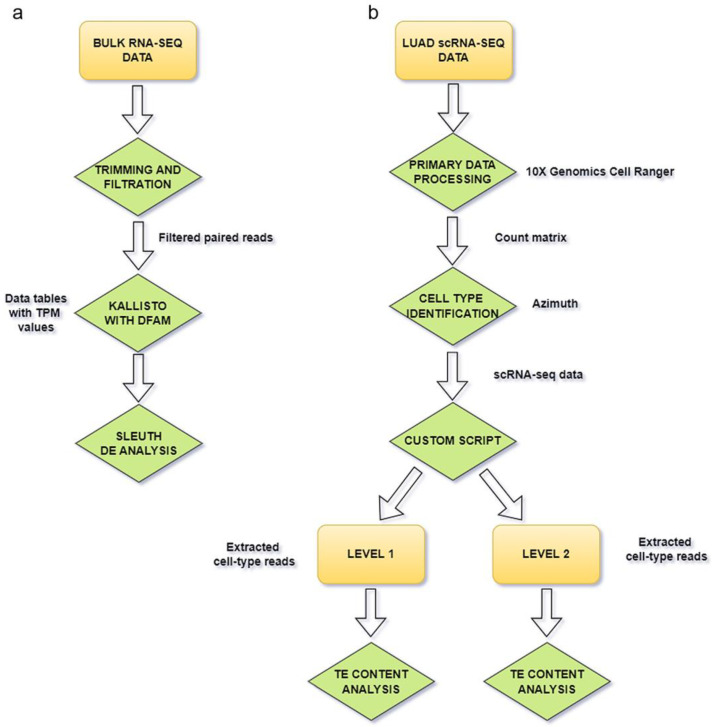
Pipelines of RNA-seq data analysis. (**a**) A scheme for bulk RNA-seq data processing, (**b**) a scheme for single-cell RNA-seq (scRNA-seq) data processing.

The pipeline of bulk RNA-seq data analysis is shown in Figure 1a. RNA-seq reads were analyzed with FastQC v0.11.5 (default settings) [27] for quality control, and then data were filtered with Trimmomatic v0.36 (default settings) [28] by cleaning residual Illumina adapters and removing low-quality bases. The alignment-free quantification tool Kallisto (v. 0.48.0, with optional arguments: --single -b 1000 -l $mean -s $std, where $mean and $std were calculated based on each cluster reads files’ content) [29] was used to measure repeats expression, and human repeat set was downloaded from the Dfam database (https://www.dfam.org/ last accessed on 5 January 2024). The sets of expressed repeats were then compared using Apache Open Office Calc (Wilmington, DE, USA)) and Venn diagrams (online source https://bioinformatics.psb.ugent.be/webtools/Venn/, last accessed on 5 March 2024). A TPM threshold greater than 1 was used to filter out expressed repeats. Differential expression of repeats was estimated using the Sleuth R package [30] (10.1038/nmeth.4324). Kallisto results used by Sleuth R package are summarized in Supplementary Table S2 (sheet “kallisto_table”).

### 2.2. Single-Cell RNA-Seq Data Processing

The single-cell transcriptomes of human LUAD, published by [31], accession numbers E-MTAB-6149 and E-MTAB-6653 (https://www.ebi.ac.uk/biostudies/arrayexpress/studies/E-MTAB-6149; https://www.ebi.ac.uk/biostudies/arrayexpress/studies/E-MTAB-6653 both last accessed on 5 January 2024), were used to study TE expression profiles in cancer cells and the TME. The single-cell RNA-seq data analysis pipeline is shown in Figure 1b. Initial read clustering and analysis were performed with 10x Genomics Cell Ranger 7.2.0 (default settings) [32]. Cell clustering was performed using the Azimuth web application (https://azimuth.hubmapconsortium.org/, accessed on 5 March 2024) and the Human Lung Cell Atlas v.2 (HLCA, [33]). Reads for 2 levels of clustering were extracted with custom script (Supplementary File S1) and used for repeats expression quantification with Kallisto (v. 0.48.0, with optional arguments: --single -b 1000 -l $mean -s $std, where $mean and $std were computed based on the contents of each cluster read file, [29]); then, the results were compared using Venn diagrams.

## 3. Results

### 3.1. Comparison of the Datasets of Expressed TEs in ESCs, Tumors, Cancer Cell Lines, and Normal Fibroblasts

**Figure 2:**
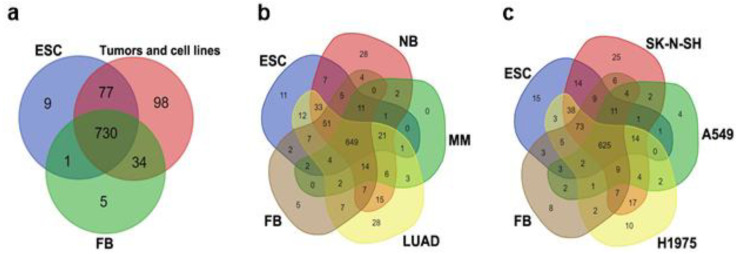
Venn diagrams illustrating comparisons of expressed TEs (**a**) between ESCs, fibroblasts, and cumulative tumor data, (**b**) ESCs, fibroblasts, and tumor tissues of different origins, and (**c**) ESCs, fibroblasts, and tumor cell lines originating from epithelial cancers (LUAD and NB). A549 and H1975—lung carcinoma cell lines, SK-N-SH—human neuroblastoma cell line, FBs—fibroblasts, NB—neuroblastoma, LUAD—lung adenocarcinoma, MM—multiple myeloma.

Transcriptomic datasets of ESCs, normal fibroblasts, tumor tissue cells, and corresponding cancer cell lines (see Materials and Methods Section) were initially qualitatively analyzed using Venn diagrams to identify both mutual and cell-type-specific TE transcripts. At this initial stage of the study, sets of Kallisto-derived expressed TE repeats were compared (Figure 2, detailed information is given in Table 2, Supplementary Table S1, and Supplementary Files S2–S4) because the majority of differential expression analysis packages employ filtering techniques, yet these methods often fail to account for transcripts that are not expressed or transcribed (i.e., with a zero value). In addition to the well-known families, rare TEs such as UCON, Eulor, Eutrep, BLACKJACK, Looper, Zaphod, Charlie, X, and Tigger were detected among the cell-type-specific repeats. BLACKJACK, Looper, Zaphod, X, and Tigger are DNA transposons, presented as fossils of ancient elements. Some X elements are non-autonomous retroelements. The terms “UCON” (ultraconserved element), “EUTREP” (eutherian repeat), and “Eulor” (euteleostomi-conserved low-frequency repeat) refer to repeats that are conserved in vertebrates but are not attributed to any of the known transposable element (TE) groups. [34]. Moreover, numerous medium reiteration frequency repeats (MERs) were identified in the datasets. Some of the MERs were retroelements or DNA transposons, while others were not attributed to any known group of TEs (shown as ‘Unknown’ in Table 2).

At the beginning of the study, the expression profile of TEs in ESCs was compared to that of normal differentiated cells (fibroblasts) and to the pooled data of the expression profiles of TEs in different tumors and cancer cell lines (Figure 2a, Table 2). Fibroblasts were taken as a representative sample of normal cells due to the following reasons: (1) the LUAD comprises a heterogeneous population of cells with diverse origins (epithelial, immune, stromal, etc.), including epithelial cells of varying types, such as vascular, lung alveolar, and airway epithelia. As a result, selecting a particular cell population to serve as an exemplar of normal cells for the construction of Venn diagrams was challenging. (2) Our study included not only epithelial tumors. MM (which has a mesenchymal origin) was also included.

A total of 730 TEs were identified that were expressed in all of the transcriptomes included in the analysis. The majority (54%) of the expressed TEs were LTR retroelements. Approximately 24% of the identified repeats were LINEs and SINEs, while DNA transposons accounted for approximately 13%, and pseudogenes occupied 7% (Supplementary File S2, Supplementary Table S1). Cell-type-specific TEs were also revealed. A list of cell-type-specific TEs corresponding to Figure 2a is given in Table 2. Notably, HERVH and other HERVs were among the TEs that were expressed in normal fibroblasts, ESCs, and cancer cells. The expression of HERV TEs was not limited to ESCs and/or cancer cells (Table S1) though the level of transcription did differ (Supplementary Table S2).

Both ESCs and tumor cells shared numerous transcriptionally active transposable elements that were silent in fibroblasts. In Venn diagrams, only 1 TE was expressed in both ESCs and in fibroblasts but not in tumor cells, while 77 TEs were transcriptionally active in ESCs, in tumor tissues, and also in cancer cell lines (Figure 2, Table 2, Supplementary Table S1). TEs that were specifically expressed in both ESCs and tumor cells were predominantly LTR elements. Additionally, the transcripts of MERs and DNA transposons belonging to the hAT (Charlie transposons) and Tigger families were identified (Table 2, Supplementary Table S1).

Tumor cells exhibited the highest number of TEs that were exclusively expressed in these cells and not present in other groups. The results showed that only 9 specific TEs were expressed in ESC, 5 TEs expressed in fibroblasts and 98 TEs expressed exclusively in tumor tissue cells and cancer cell lines (Figure 2a). These findings confirm that TE transcription is dysregulated in cancer cells and tumor tissue compared to normal stem (ESC) and differentiated (fibroblasts) cells. Retroelements were the dominant TE group among those selectively transcribed in tumor tissue and cancer cells. (Table 2, Supplementary Table S1).

ESCs had a small number of specific repeats, which were represented by LTR elements (LTR33A, LTR82B, MLT2E, MER110A) and DNA transposons of the hAT and Tigger families (Arthur1C, Charlie14a, Tigger13a, Tigger17b, Tigger8, EutTc1-N1, MER121B, MER63A, X6a_DNA).

The establishment of primary (i.e., tissue-derived) cell cultures is accompanied by genome reorganization and epigenetic reprogramming that both contribute to genomic instability. TE activation and transcription are influenced by genetic instability and inter-individual differences [35,36,37,38,39,40]. Therefore, the next step was to determine whether the expression profile of TEs in a tumor differs from the expression profile in a cancer cell line derived from the tumor. At first, the ESC datasets were compared separately to tumor datasets and cancer cell line datasets (Figure 2b,c; Supplementary Table S1). The number of TEs expressed in each comparison group was similar regardless of the datasets employed for comparison: e.g., 649 and 625 TEs were expressed in tumor tissues and cancer cell lines, respectively (Figure 2b,c and Supplementary Files S3 and S4). However, each of the datasets (ESCs, fibroblasts, tumors, and their corresponding cell lines) had a unique signature of expressed TEs (Supplementary Table S1). In tumor tissues and in cancer cell line transcriptomes, the TEs detected were also expressed in ESCs. Some TEs, such as DNA transposons Riksha, LINEs L1M4a1_5end, and L1MEi_5end, as well as LTR/ERVs—LTR2752, LTR53B, MLT1F-int, and MER67D—were found in all three groups (ESCs, tumor tissues, cancer cell lines) (Table 3, Supplementary Table S1). These sequences may play a role in cell proliferation or stemness maintenance.

**Figure 3:**
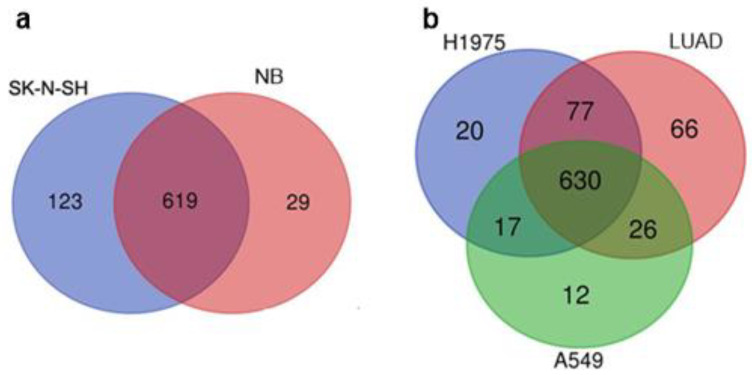
Venn diagrams illustrating the comparison of TE expression patterns between tumor tissues and corresponding cancer cell lines. (**a**) Neuroblastoma (NB) vs. SK-N-SH cell line, (**b**) lung adenocarcinoma (LUAD) vs. H1975 and A549 cell lines. A549 and H1975—LUAD cell lines, SK-N-SH—a human NB cell line.

The tumor transcriptomes analyzed in this study were obtained from whole tumor tissues, which consist of both cancer cells and non-malignant cells of the TME [22,23]. Therefore, we compared the TE expression patterns of tumors and their corresponding cancer cell lines (SK-N-SH cell line vs. NB and A549, H1975 cell lines vs. LUAD) (Figure 3). Overall, the majority of the expressed TEs (>600) were the same in both the cell lines and the corresponding tumors. Nevertheless, some transcripts of TEs were only identified in bulk tumor tissue or in the corresponding cancer cell line. (Figure 3, Table 3, Supplementary Files S5 and S6).

The SK-N-SH neuroblastoma cell line exhibited the most diverse set of specific repeats, primarily represented by DNA transposons and LTR/ERV retroelements. Additionally, this cell line expressed the highest number of specific pseudogenes. In contrast, when compared to LUAD cell lines, the LUAD tumor displayed a greater diversity of specific elements. The A549 datasets contained more pseudogenes but fewer ERV\LTR and DNA transposon RNAs compared to the H1975 cell line datasets (Table 3, Supplementary File S6). The discrepancies observed were likely attributable to the fact that tumors are composed of a heterogeneous population of cells, including both malignant and non-malignant elements. This heterogeneity may have contributed to the observed variations in the composition of the bulk datasets.

Thus, the analysis of the data sets using logical operations (Venn diagrams) revealed the existence of TEs that are common to both ESCs and cancer cells, as well as TEs that are unique to each type of tumor.

### 3.2. Differential Expression of TE Transcription in ESC, Normal Fibroblasts, Tumors, and Cell Lines

**Figure 4:**
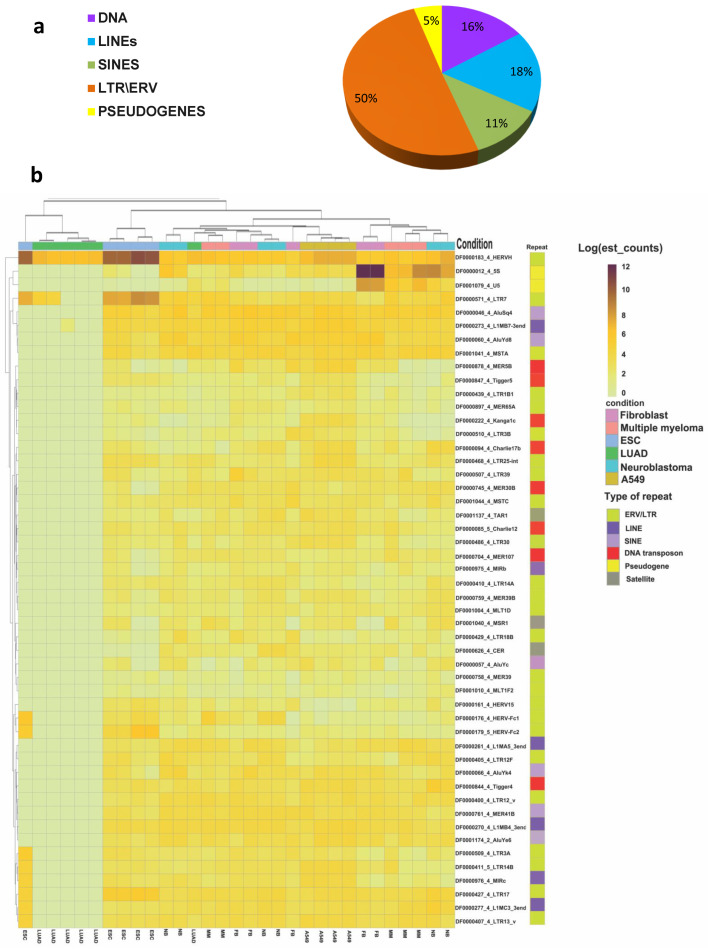
Analysis of TEs differential expression. (**a**) The percentage of differentially expressed transposable elements (TEs) (DNA transposons, LINEs, SINEs, LTR/ERVs, and pseudogenes). (**b**) Heatmap based on log(TPM) of the top 50 differentially expressed repeats. The colors above the heatmap indicate the cell type, while the colors on the right of the heatmap indicate the type of repeat. Clusterization cladograms are also shown near the heatmap. LUAD—lung adenocarcinoma, MM—multiple myeloma, NB—neuroblastoma, ESCs—embryonic stem cells. TE IDs in (**b**) are shown as DfamID_TE name.

**Figure 5:**
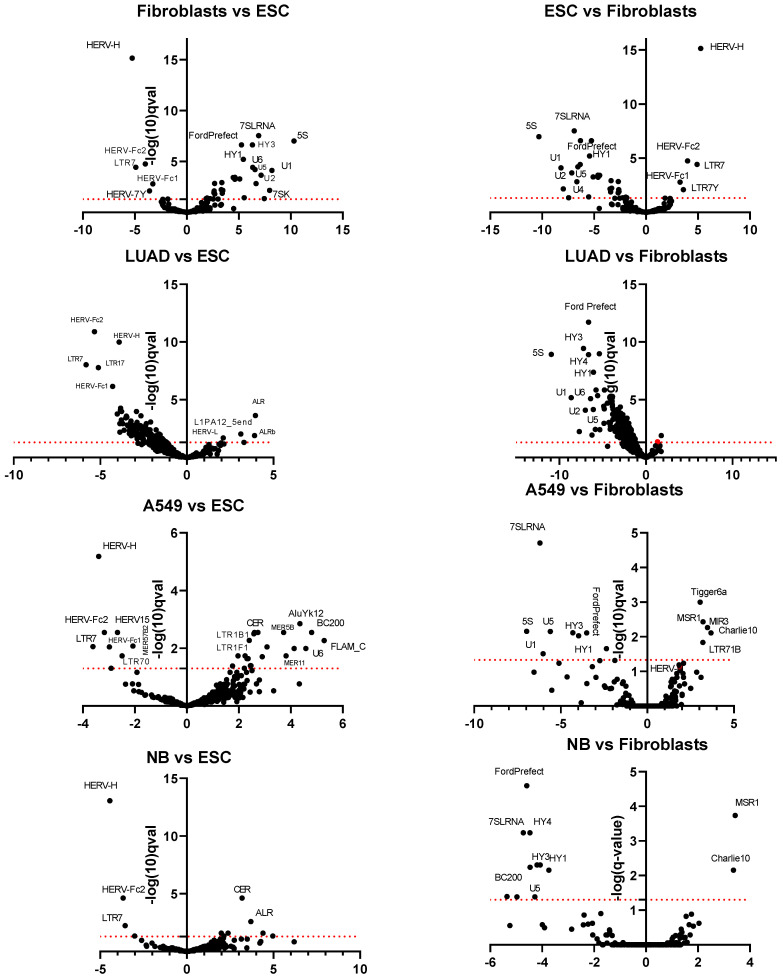
Volcano plots of differentially expressed TEs in normal fibroblasts, lung adenocarcinoma (LUAD), NB (neuroblastoma (NB), and LUAD cell line A549 vs. embryonic stem cells (ESCs) (*left column*) or in ESCs, LUAD, NB, A549 vs. fibroblasts (*right column*). *X*-axis—*b* or *beta*-value (log_2_ fold changes between conditions) calculated by the Sleuth package. *Y* axis—lࢤog(q-value); the red dotted line corresponds to q-value < 0.05, the dots above the line are either up-regulated (b < 0) or downregulated (b > 0). The red dot in the LUAD vs fibroblasts plot corresponds to HERVH.

The initial phase of the study employed the plotting of Venn diagrams (Figure 2 and Figure 3), a qualitative data analysis approach that employs logical operators and is useful for roughly estimating the differences between data sets. To quantify the difference in TEs expression and evaluate the statistical significance, differential expression analysis was performed, revealing 243 repeats with significant changes (q-value ≤ 0.05, where q-value is an adjusted p-value found using an optimized false discovery rate, FDR, approach) (Supplementary Tables S1 and S2 and Figure 4a and Figure 5). LTR retroelements accounted for 50%, LINE and SINE for 29%, and DNA transposons together with pseudogenes for the remaining 21% of differentially expressed TEs (Figure 4a). A heatmap of the top 50 differentially expressed repeats (i.e., repeats with the minimal *p*- and q-values as calculated by the Sleuth package) was constructed using hierarchical clustering (Figure 4b).

In all samples of LUAD, transcription of most TEs was repressed compared to other groups (Figure 4b). Nevertheless, transcription of some LTRs was at the same level as in other non-ESC transcriptomes (Figure 4b). It is also noteworthy that the clustering of samples does not always align with the expected cell type. Only samples of A549 cells formed a homogeneous group. A single LUAD sample was included in the MM group, while another ESC sample was segregated with the LUAD. Additionally, samples of fibroblasts, MM, and NB cells formed mixed groups in various combinations. This phenomenon may be attributed to a number of factors, including inter-individual differences and variations in the cellular composition of tumor samples. In the heatmap, only a few TEs (HERVH and LTR7, as well as pseudogenes U5 and 5S) exhibited increased expression levels in LUAD. The up-regulation in fibroblasts in comparison with other cells was confirmed by the Wald test (Figure 5, Supplementary Table S2). The slight increase in HERVH transcription in LUAD in comparison with fibroblast transcriptomes was statistically significant (Figure 4 and Figure 5, Supplementary Table S2).

Differential expression analysis also allowed for identifying common features in the transcriptional profile of ESCs and tumor cells and revealing candidates for tumor-specific TEs. Tumors and ESC transcriptomes were compared against those of fibroblasts to assess the differences between ESCs and tumor cells from non-malignant somatic cells. Additionally, a comparative analysis of fibroblasts and tumor transcriptomes with ESC transcriptomes was conducted to evaluate the similarities and differences between cancer cells and ESCs (Figure 5). Four TE RNAs (HERVH, LTR7, HERV-Fc1, HERV-Fc2) were significantly downregulated in both fibroblasts and all cancer tissues and cell lines when compared with ECSs (Figure 5). HERVH and LTR7 RNA (stand-alone copies of HERVH-flanking regions containing viral regulatory elements) are involved in the regulation of the pluripotency state and are up-regulated in ECSs [41,42,43]. HERV-Fc1 and HERV-Fc2 are included in the enlarged ERV-F/H family [44], but their role in pluripotency regulation has not been confirmed.

In all other parameters, the TE transcription profiles of NB and MM did not differ significantly from the ESC TE RNAs profile (Figure 5, Supplementary Figure S1). The up-regulated lncRNAs (CERs—centromeric repeats, ALRs—alphoid repeats) with the highest qval value (i.e., the most significant difference) belonged to tandemly repeated DNA. The situation was different for LUAD transcriptomes. Transcription of TEs was generally downregulated in this tumor tissue as compared to ESCs (Figure 4b and Figure 5; Supplementary Table S2). Nevertheless, in A549 cells originated from LUAD, TEs were not downregulated to the same extent as in LUAD. The TE RNAs profile of A549 was closer to that of ESCs and other tumor tissues (excluding LUAD). It is unclear whether this is due to the TME background in the LUAD transcriptomes or the long-term in vitro expansion of A549.

A comparison of transcriptomes between ESCs, cancer cell lines, and tumor tissues versus normal fibroblasts allowed us to reveal common features of TEs transcription in cancer (Figure 5, Supplementary Table S2). The retropseudogenes derived from the human Ro/SS-A autoantigen-associated hY RNAs (HY1, HY3, HY4) were downregulated in all cancer samples but not in ESCs. The pseudogenes for the small nuclear RNAs (snRNAs U1, U2, U3, U4, U5, and U6) were downregulated in both cancer samples and in ESCs. Notably, transcription of the FordPrefect DNA transposon of the hAT-Tip100 family was downregulated in all analyzed groups of data sets (ESCs, tumor tissues, cell lines) compared to fibroblasts. The Charlie10 DNA transposon, on the other hand, was up-regulated in NB and A549 cells. Only one differentially expressed repeated sequence, a minisatellite repeat, MSR1, was up-regulated in all cancer samples but not in ESCs (Figure 5). However, differential expression analysis can be obscured by the recombination and copy number expansion that is characteristic of minisatellites.

The differential analysis data indicate that the up-regulation of HERVH, LTR7, HERV-Fc1, and HERV-Fc2 RNAs is a feature of ESCs. The downregulation of HY scRNA pseudogene transcription in conjunction with MSR1 RNA up-regulation might be a feature of tumor tissues. The initial qualitative logical analysis (Venn diagrams) of the data yielded evidence suggesting the existence of additional cancer-specific TEs.

### 3.3. TE Expression in Cancer Cells and in the TME

**Figure 6:**
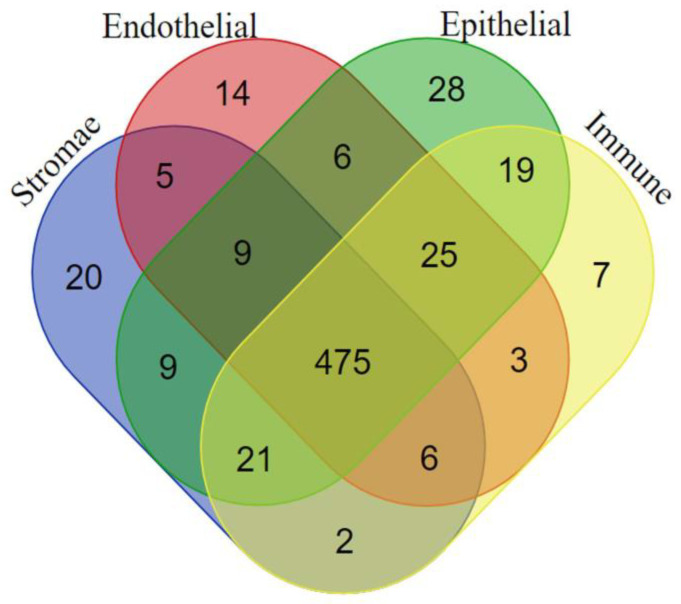
A Venn diagram illustration of TEs expressed in LUAD at the first level of cells clustering when cells are clusterized as stromal (fibroblasts and mesenchymal stromal cells or MSCs), endothelial, epithelial, and immune.

Our study demonstrated the difference between TE transcriptional profiles in tumors and corresponding cancer cell lines (Table 3; Figure 3 and Figure 5). A tumor is not a homogeneous formation of cells. In tumors, a specific tissue structure, the TME, is formed that supports the life of the tumor. The TME is composed of cells of epithelial, endothelial, and stromal origin [22,23]. With the single-cell RNA sequencing technology, it is now possible to separate transcriptomes of different cell populations in tissues. To interpret the data generated by single-cell sequencing, computational methods of data clustering are employed to identify cell populations. The goal of clustering is to detect distinct cell populations that can be annotated as known cell types or discovered as novel ones. Clustering can be performed at different levels of resolution, i.e., different degrees of detail. In our analysis of LUAD single-cell sequencing data published in [31], two levels of resolution were used for TE transcription analysis. Figure 6 shows a comparison of cells at the first (i.e., less detailed) level of clustering.

At this level, LUAD tumor cells were classified into four tissue types: stromal (fibroblasts and mesenchymal stromal cells or MSCs), epithelium (including cancer cells), endothelium, and immune (Figure 6). More than 470 TEs were transcriptionally active in all tumor cells analyzed, confirming a general deregulation of the TEs transcriptional pattern (Supplementary File S7). The TEs transcription pattern was diverse and included both class I and II TEs: ERV (with HERV-H among them), LTR, Alu, Tiggers, etc. Each tissue type exhibited a specific pattern of expressed TEs. The data on TE patterns are summarized in Table 4 and Supplementary File S7. The sets of TEs that were unique to each cell type were predominantly composed of LTR retroelements. The stroma and epithelium (with cancer cells) had the highest number of expressed TEs (Table 4). The cells of both clusters expressed HERV-Fc2, while epithelial and cancer cells additionally expressed HERV-Fc1 (Table 4 and Supplementary File S7). Given the data indicating a strong up-regulation of these TEs in ESCs (Figure 5) but not in normal tissues, it can be proposed that their deregulation is a component of carcinogenesis.

Transcripts of TEs in TME immune cells were represented exclusively by ERV\LTR retroelements. However, one of them, LTR48 was also expressed in ESCs and some tumors (Table 4).

A detailed list of uniquely expressed elements for the cells at the second (more detailed) level of clustering is shown in Table 5 and Supplementary File S8. Cells of the LUAD airway epithelium (EP in Table 5; includes both cancer cells and the normal epithelium) had the most diverse pattern of TEs expression. Most of the TEs in EP were classified as ERV\LTR retroelements. One detected LTR retroelement, MER73, was found only in airway EP. Some of the detected TE RNAs were identified in ESCs and whole LUAD tumor bulk-transcriptomes: MER66-int, LTR26B, LTR21C, HERVK11D, and Rep522. The TEs that were detected earlier in the study of whole tumor tissue transcriptomes (Table 2)—LTR52-int, L1PA17_5end ¬—were also found in EP cancer cell datasets. Alveolar EP contains two elements that match the sets of ESCs and tumors—MER192-int and tumors—MER89; LTR18A was found only in alveolar cells.

The pattern of TEs expression in the LUAD TME fibroblasts largely differed from the one observed in normal fibroblasts (Table 2, Table 5 and Table S1, Supplementary Files S7 and S8). UCON23 (also found in the stroma at the first stage of clustering—Table 4) and L2b_3end transcripts were detected only in TME cells of fibroblast lineage, including cancer-associated fibroblasts (CAFs); L1MEg_5end and Ricksha elements were transcribed in ESCs and tumors. The remaining elements were identified in the total pool of elements expressed by tumors, fibroblasts, and ESCs.

Lymphoid and mesothelial cells did not express any tumor or ESC-specific TEs, whereas myeloid cells contained the MLT1G transcript, which was specific to ESCs and tumor transcriptomes but was not detected in cancer cell line transcriptomes. Blood vessel cells expressed specific DNA transposons, retrotransposons, and unclassified elements, with L1MEb_5end, LTR37B, and Tigger5b being characteristic of ESCs and tumors, UCON9 and UCON64 specific for tumors, and LTR16A2 found in normal fibroblasts.

Thus, most of the TEs were transcribed in all tissues and cell lines used in the study while some subsets were specific for cancer cells or cells of the TME. Cancer cells share, to some extent, the TEs expression pattern with ESCs.

## 4. Discussion

TEs are known to act as genome-regulatory elements, influencing gene transcription, splicing, and genome architecture [9,45,46,47,48,49,50]. Despite the presence of transcripts derived from TEs in human RNA-seq data, the meaning of this phenomenon has been largely overlooked for an extended period of time as it has been assumed that TEs are exclusively transcribed in the germline cells, placenta, and preimplantation embryo. The TEs transcription is derepressed in ESCs and preimplantation embryos and declines rapidly at the end of the blastocyst stage [9,14,51,52,53,54,55]. Nevertheless, full-length and partial transcripts of transposons have previously been found in somatic cells, with a large variation in transcription levels between tissue types [56,57]. The biological roles for some of the TE transcripts are well documented [9,53,58,59], while the functions of others remain to be elucidated. Three hypotheses are now discussed; probably, all of them are correct. 1. The majority of TEs are repressed in somatic tissues, yet their activation initiates the transposition process. Such transposon activity has the potential to disrupt gene expression and function by inserting into the promoter or coding sequence. 2. The activation of TE promoters results in the activation of oncogene expression (onco-exaptation). 3. Tumorigenesis can be conceptualized as a “funhouse mirror” of embryogenesis. It is established that the epigenetic reprogramming of the extra-embryonic lineage mirrors the somatic transition to cancer. Dedifferentiation is recognized as a hallmark feature of cancer cells. This enables a resulting phenotype of proliferation, self-renewal, and a metabolism reminiscent of embryonic stem cells [19].

### 4.1. The Transcription Profile TEs in Tumor Tissue Is Similar to ESCs with the Exception of Four TEs

According to our data, cancer cells or tumor tissues generally expressed the largest set of TEs (Figure 2, Table 2, Supplementary File S1). TEs are transcriptionally silent under strict epigenetic regulation after implantation but can potentially be reactivated by malignant transformation [9,50,60]. These reactivated TEs are involved in chromatin remodeling, alternative splicing, interaction with the immune system, and many other processes. These are normal functions of TE transcripts; however, when activated at an inappropriate time, they may lead to malignant transformation [11,50,53,61,62,63]. A qualitative analysis (Venn diagram) revealed 77 TEs transcribed in both ESCs and tumor tissues or cancer cell lines (Figure 2, Table 2). No TEs were up-regulated in tumors as compared with ESCs. It was an unexpected finding that the up-regulated repeats belonged to different families of tandemly repeated DNA but not to TEs (Figure 5).

The loss of differentiation markers in tumors and the subsequent reacquisition of an epigenetic landscape reminiscent of early developmental stages has been well documented [19,64]. Cancer cells (especially cancer stem cells) share some characteristics with ESCs and induced pluripotent cells: replicative immortality, increased proliferative capacity, expression of OCT4, NANOG, and KLF4 (factors that induce cellular reprogramming, or are fundamental for maintaining a pluripotent state and are also potent oncogenes), and telomerase activation [19,65]. Considering the partial reactivation of pluripotency genes in cancer cells, the reactivation of TEs involved in maintaining the activity of pluripotency genes is predictable. HERVH was most actively transcribed in ESC samples, followed by LUAD. NB and MM tissues were also enriched for HERVH transcripts, but to a lesser extent. The lowest number of HERVH transcripts was detected in fibroblast transcriptomes (Supplementary Table S2). The precise role of HERVH in the process of carcinogenesis remains unclear. One hypothesis suggests that its activation is part of a general dedifferentiation program [19]. HERVH involvement in TAD formation [55] indicates that this TE may contribute to the formation of new topological domains and, consequently, epigenetic reconfiguration of the genome. Additionally, HERV-encoded sequences are considered as a new class of tumor-specific antigens [66].

In differential expression quantitative analysis (Figure 4 and Figure 5), four TE RNAs (HERVH, LTR7, HERV-Fc1, HERV-Fc2) were significantly up-regulated in ESCs. This set of four transcripts constituted the most striking and distinguishing feature of the ESC TEs transcription profile. The up-regulation of HERVH transcription in ESCs is well documented [58,67,68,69]. The role of HERVH in the maintenance of pluripotency, probably through the establishment of topologically associated domain boundaries, has been demonstrated [58,70]. Lu et al. (2014) reported that LTRs function as enhancers and that HERVH is a nuclear lncRNA required to maintain human ESC identity [55]. The authors proposed that HERVH interacts with coactivators and pluripotency factors such as OCT4 to promote the enhancer activity of LTR7 (an LTR flanking many HERVH insertions) and nearby regions cobounded by p300 and OCT4 to drive the expression of neighboring lncRNAs and protein-coding genes essential for human ESC identity [55]. HERVH-derived transcripts are now considered as a hallmark of human pluripotent stem cells. Their transcription is also a critical mechanism for induced pluripotent stem cells formation [71]. Active copies of HERVH have binding sites for four key transcription factors that drive pluripotency, such as OCT4, SOX2, LBP9 (TFCP2L1), and NANOG [69]. HERVH was transcribed in all the samples analyzed in the study though its transcriptional activity was variable. (Table 2 and Table S1, Figure 4b and Figure 5). The highest level of transcription was detected in ESC samples followed by LUAD cell line A549 and LUAD samples (b-value for A549 HERVH RNA vs. ESC—−3.4, LUAD—−3.9 vs. −4.4 and −5.3 for NB and MM). In all LUAD tissue samples HERVH was one of the few TEs expressed at a relatively high level, although LUAD was characterized as having the lowest level of TEs expression among the samples studied (Figure 4b). HERVH transcription was slightly up-regulated compared to normal cells (Figure 5, Table S2). The TE was expressed in some cancer cell lines, including A549 [72], and was most abundant in LUAD tumor [73]. We consider HERVH as a potential diagnostic marker and an LUAD therapy target.

LTR7 is the regulatory elements for HERVH. However, the recombination of ERVs leads to the appearance of stand-alone copies of LTRs in the genome. The transcription of these solo-LTR7 copies is also highly up-regulated in ESCs of the inner cell mass. Nevertheless, the functions of LTR7 RNA in pluripotent cells identity maintenance remain unknown. [42,51,58,74]. However, it is correlated with the appearance of topologically associated domain boundaries in primate pluripotent stem cells [43].

Two other TEs of the retrotransposon group were found to be significantly up-regulated in ESCs: HERV-Fc1 and HERV-Fc2 (Figure 5). The HERV-Fc family is closely related to the HERV-H family and is included in the expanded HERV-H/F family [44,75]. HERV-Fc1 was identified with a full-length coding envelope gene in primates and is now considered to be one of the most intact HERV viruses [44]. Its transcription in ESCs is higher than in blood mononuclear cells but much lower than in some blood malignant disorders [67]. The ERV is also up-regulated in СD4+ and CD8+ lymphocytes in patients with active multiple sclerosis [76].

Each of the TEs up-regulated in ESCs can be transcribed in other tissues. However, when actively transcribed together, they may be considered as a TE markers set for ESCs. The up-regulation of HERV-Fc1 and HERV-Fc2 as described in our study suggests their potential role in early embryogenesis.

### 4.2. The Up-Regulation of Tandem Repeats and Downregulation of Pseudogenes Transcription in Tumors

Differential expression analysis revealed 243 TEs with significant changes in expression between different cell types. Most of these TEs have been described as differentially expressed for the first time. The finding opens up a vast field of potential research and ideas. To elucidate their function, further in vitro and in situ studies are essential. The up- or downregulation of the majority of these differentially expressed TEs will be confirmed in vitro in further studies.

The repeat expression profiles of LUAD, MM, and NB exhibit both similarities and differences. The LUAD TEs expression profile is distinctive when compared to other cancers. The majority of the differentially expressed TEs are observed to be downregulated in LUAD (Figure 4). However, this is not the case for HERVH. In our view, this finding highlights the significance of HERVH in the process of carcinogenesis.

The differences between samples of the same cancer can be a result of varying tumor microenvironments (TMEs) and a diverse proportion of the TME and cancer cells.

The only repetitive sequence activated in all tumor samples but not in ESCs when compared to fibroblasts was a minisatellite repeat MSR1 (Figure 5). It is a 36–38 bp minisatellite sequence specific to chromosome 19 [77]. In the genome, MSR1 repeats are global regulators of gene expression in breast and prostate cancer [78]. Its expression has been demonstrated in acute myeloid leukemia [79]. The appearance of its RNA in solid tumor transcriptomes and in MM has been shown for the first time in the current study (Supplementary Figure S1). Recently, minisatellites (short tandem repeats, STRs) have been established as transcription start sites (TSSs). It is known now that the transcription of many mRNAs starts not from a promoter site but from a TSS. Thousands of STRs can initiate transcription in human and mouse [80]. Genetic variants linked to human diseases are preferentially found at STRs with a high transcription initiation level, supporting the biological and clinical relevance of transcription initiation at STRs. Copy number variation (CNV) in these sequences is an important regulator of the genes controlled by them. The methods employed in our study did not distinguish an increase in transcriptional activity and an increase in length of the transcribed MSR 1 STR. However, MSR1 is a minisatellite repeat. Minisatellites are prone to copy number variation, which may affect differential expression analysis of transcriptomes. An assessment of the MSR1 copy number in different cell lines would be helpful to verify its up-regulation in cancer. If verified, the finding allows for the development of tools for screening, diagnosis, and prognostication.

The transcription of tandem repeats distinguished tumor tissues from ESCs as well (Figure 5). The up-regulation of pericentromeric big satellites transcription in cancer has been demonstrated [25,54]. However, the transcription of centromeric ALR and CER in cancer has not yet been reported.

Notably, transcription of the FordPrefect DNA transposon of the hAT-Tip100 family was downregulated in all analyzed groups of data sets (ESCs, tumor tissues, cell lines) compared to fibroblasts. This DNA transposon belongs to the HAT-tip100 family [81] but little is known about its functions. Our data suggest it as being responsible for some specific functions in fibroblasts.

The retropseudogenes derived from the human Ro/SS-A autoantigen-associated hY RNAs (HY1, HY3, HY4) were downregulated in all cancer samples but not in ESCs when compared with fibroblasts (Figure 5). These pseudogenes are L1-dependent non-autonomous retroelements, potentially involved in the post-transcriptional regulation of gene expression [82]. The pseudogenes for the small nuclear RNAs (snRNAs U1, U2, U3, U4, U5, and U6) exhibited decreased expression in both cancer samples and in ESCs, thereby providing further evidence of a shared feature between ESCs and tumor cells.

Thus, in silico studies have revealed that up-regulation of tandem repeats transcription (especially MSR1) and downregulation of some pseudogenes (such as HY pseudogenes) are specific for tumors. To corroborate the in silico results, the level of their transcription should be measured in in vitro studies (qPCR). These data must be contextualized with the patients’ medical history records, including age, treatment, tumor grade, treatment outcome, and comorbidities.

These repeats and pseudogenes were differentially expressed. However some other TEs listed in the Table 2 (mostly ERV and LTR) were classified as unique for some datasets during qualitative analysis that employ logical operators. Many of them have not yet been implicated in any process of pluripotency or malignancy maintenance and have not been previously described in cancer tissues (e.g., CR1Amni-1, CR1-16AMi, L1M2a1_5end, L1M7_5end, L1M8_5end, L1MCc_5end, etc.). Therefore, these elements are promising candidates for use as prognostic markers or therapeutic targets.

The difference between the TE expression profiles of tumor tissue samples and cancer cell lines is reported here (Figure 3, Table 3). Rewiring of epigenetic marks (histone modifications, DNA methylation) during adaptation of cells to in vitro conditions has been reported [39,40]. This epigenetic reprogramming is likely to be one of the major factors involved in the activation of TEs. It imposes certain limitations on the use of in vitro models for cancer research. The difference in the transcriptional patterns of TEs between tissue cells and expanded cells or cell lines should be taken into account when choosing an experimental model or translating the results.

### 4.3. The Transcription Profile of TEs in Cancer Cells and the TME

The interactions of the various cell types within a tumor cooperatively create a supportive niche (the TME) that promotes cancer cell survival, proliferation, and evasion from immune surveillance [83,84,85].

The transcription of TEs is activated in the whole tumor. A total of 475 TE transcripts were identified during the analysis of single-cell sequencing data (Figure 6). The role of a limited number of TEs in cancer has been demonstrated in previous studies [15,19,63,67]. Our findings provide evidence of general deregulation and highlight potential new targets for further investigation and anti-cancer therapy.

We analyzed the Human Atlas data on LUAD single-cell sequencing. The Human Lung Atlas provides a detailed high-resolution reference of the lung’s cellular and molecular composition, but it has limitations. The sample diversity is limited, potentially not capturing the full genetic, environmental, and lifestyle variability of the broader population. Additionally, certain diseases may be under-represented, and technological variations in single-cell RNA sequencing can introduce biases. Integrative efforts across different datasets can complicate data harmonization. We understand these limitations; however, the primary screening data clearly delineate differences, and these methodological constraints do not affect our conclusions. The in silico data reported here confirmed the difference in TE expression profiles between LUAD cancer cells and the TME cells (Figure 6, Table 4 and Table 5).

Each cluster (i.e., an annotated cell population) has specific TE-derived RNAs. The stromal cells (fibroblasts and MSCs) and the LUAD airway epithelial cells exhibited the greatest number of specific TE RNAs (Table 5), with the majority of these being retroelements. LUAD airway epithelial cells expressed HERVK11D, a member of the HERVK family known to be expressed by cancer cells in tumors [86,87,88]. The functions of other LUAD epithelial TE RNAs (Table 4 and Table 5) have not yet been reported. In cancer-associated cells of fibroblast lineage, 11 TEs were specifically expressed (Table 5). One of them, MLT1I, has been reported to be transcriptionally activated in response to interferon-γ stimulation in small-cell lung cancer cell lines that have undergone mesenchymal transformation [89]. Cancer-associated MSCs (including cancer-associated fibroblasts) respond to interferon-γ, which plays a key role in altering the function of cancer-associated stromal fibroblasts [90,91]. Whether or not MLT1I is involved in this pathway remains to be investigated. HERV-Fc1 LTR2 TEs were also specifically expressed in cancer-associated fibroblasts (Table 5). HERV-Fc1 TEs belong to the HERV-H/F family, which is known to be involved in tumor progression [63]. Our data indicate that it is up-regulated in ECSs.

Our analysis of transcriptomes revealed TEs specifically transcribed in the TME. It is a highly complicated structure sometimes referred to as a “tumor ecosystem” that contains non-cancer cells (vessel cells, stromal, immune cells, etc.) and various cytokines and chemokines secreted by them. The interaction between tumor cells and the TME contributes to carcinogenesis, metastasis, and drug resistance. Non-coding RNAs are involved in cancer development through targeting the cellular components of the TME [24,25,92]. Non-coding RNAs modulate and are modulated by numerous signaling pathways (such as the WNT-, MAPK, PI3K/AKT, STAT3, Notch, p53, NER, NF-kB) in cancer cells and the TME [24]. TE RNAs are a part of this orchestra of ncRNAs. TEs have been shown to be a key player in immune regulation in solid tumor [53]. Their high level is associated with poor prognosis in patients with a high immune infiltration of solid tumor, which, in general cases, is associated with a good prognosis. In the patient group referred to as “immune overdrive” patients, TEs activate the IFN-mediated inflammatory pathway in immune cells but not in tumor cells, triggering in immune cells the expression of PD-L1 and thus promoting immune evasion [93].

We found that some of the ERVs that are strongly up-regulated in ESCs are expressed in tumors: HERVH, HERV-Fc1, and HERV-Fc2. The analysis of single-cell transcriptomes revealed that HERVH was transcribed both in cancer cells and the TME: HERV-Fc2 in stromal fibroblasts and epithelial and cancer cells, while HERV-Fc1 was specific for the cluster of cells that included only epithelial and cancer cells (Figure 6, Table 4 and Supplementary File S8). The transcription of these TEs during carcinogenesis has been demonstrated in previous studies [63,67]. Our findings indicate, for the first time, that they are expressed at different levels in the TME and cancer cells, suggesting that they may have distinct roles in tumor progression. We plan to further validate these findings in future in vitro studies.

## 5. Conclusions

In this study, the strong up-regulation of four TE RNAs in ESCs was demonstrated. The up-regulation of two of these transcripts (HERV-Fc1 and HERV-Fc2) was established for the first time, indicating a more extensive involvement of TEs in the maintenance of pluripotency than was previously assumed. The general deregulation of TEs transcription in tumors of diverse origin was demonstrated. The deregulated transcription profile exhibited similarities to that of ESCs. Derepressed TEs are present in somatic tissues in a limited quantity, although, in tumors, many dormant TEs are activated with an expression pattern similar to ESCs to some extent. However, in tumors, TEs are expressed not only in cancer cells but also in cells of the TME, especially in those of fibroblast origin (including cancer-associated fibroblasts). Our study provides new data on the transcriptional profile of TEs. Whether the differentially expressed TEs reported here can be used as prognostic markers or markers of the TME or cancer cells of a specific origin can be evaluated in future investigations. The TE-derived RNAs revealed in the present study may be involved in TME remodeling and innate immune response, opening avenues for novel approaches to cancer therapy and tumor microenvironment profiling.

## Figures and Tables

**Table 1:** RNA-seq data, used in the study.

SRA	Cell Type	Cell Line	Instrument	Selection	Layout	Analysis *
SRR11802228	Undifferentiated hESCs	WA09	Illumina HiSeq 4000	cDNA	SINGLE	Venn/DE
SRR11802260	Undifferentiated hESCs	WA09	Illumina HiSeq 4000	cDNA	SINGLE	Venn/DE
SRR19763997	Human embryonic stem cells (hESCs),	H9	Illumina HiSeq 4000	cDNA	PAIRED	Venn/DE
SRR19763998	Human embryonic stem cells (hESCs)	H9	Illumina HiSeq 4000	cDNA	PAIRED	Venn/DE
SRR19763999	Human embryonic stem cells (hESCs)	H9	Illumina HiSeq 4000	cDNA	PAIRED	Venn/DE
SRR17011020	Human neuroblastoma cells	-	Illumina NovaSeq 6000	cDNA	PAIRED	Venn/DE
SRR17011019	Human neuroblastoma cells	-	Illumina NovaSeq 6000	cDNA	PAIRED	Venn/DE
SRR26588672	Human neuroblastoma cells	SK-N-SH	Illumina NovaSeq 6000	cDNA	PAIRED	Venn/DE
SRR26588673	Human neuroblastoma cells	SK-N-SH	Illumina NovaSeq 6000	cDNA	PAIRED	Venn/DE
SRR21143000	Human neuroblastoma cells	-	Illumina HiSeq 2000	cDNA	PAIRED	Venn/DE
SRR21142995	Human neuroblastoma cells	-	Illumina HiSeq 2000	cDNA	PAIRED	Venn/DE
SRR24600381	Lung adenocarcinoma	H1975	Illumina HiSeq 2500	cDNA	PAIRED	Venn/DE
SRR24600382	Lung adeno-carcinoma	H1975	Illumina HiSeq 2500	cDNA	PAIRED	Venn/DE
SRR24600383	Lung adeno-carcinoma	H1975	Illumina HiSeq 2500	cDNA	PAIRED	Venn/DE
SRR24166172	Lung adeno-carcinoma	-	Illumina NovaSeq 6000	cDNA	PAIRED	Venn/DE
SRR24166174	Lung adeno-carcinoma	-	Illumina NovaSeq 6000	cDNA	PAIRED	Venn/DE
SRR24166176	Lung adeno-carcinoma	-	Illumina NovaSeq 6000	cDNA	PAIRED	Venn/DE
SRR12924510	Multiple myeloma	-	Illumina NovaSeq 6000	cDNA	PAIRED	Venn/DE
SRR12924509	Multiple myeloma	-	Illumina NovaSeq 6000	cDNA	PAIRED	Venn/DE
SRR2497378	Multiple myeloma	-	Illumina HiSeq 2000	Random	PAIRED	Venn/DE
SRR2497389	Multiple myeloma	-	Illumina HiSeq 2000	Random	PAIRED	Venn/DE
SRR2497395	Multiple myeloma	-	Illumina HiSeq 2000	Random	PAIRED	Venn/DE
ERR1406030	Lung adenocarcinoma	A549	Illumina HiSeq 2500	cDNA	PAIRED	Venn/DE
SRR15410445	Lung adeno-carcinoma	A549	Illumina HiSeq 2500	cDNA	PAIRED	Venn/DE
SRR15410446	Lung adeno-carcinoma	A549	Illumina HiSeq 2500	cDNA	PAIRED	Venn/DE
SRR15410447	Lung adeno-carcinoma	A549	Illumina HiSeq 2500	cDNA	PAIRED	Venn/DE
SRR26713630	Fibroblast, primary cells from lung, 2D culture	-	Illumina NovaSeq 6000	cDNA	PAIRED	Venn/DE
SRS9826564	Fibroblast, untreated normal cells	-	NextSeq 500	cDNA	PAIRED	Venn/DE
SRS9826571	Fibroblast, untreated normal cells	-	NextSeq 500	cDNA	PAIRED	Venn
ERR12530401	Fibroblast, normal cells	-	Illumina HiSeq 2500	PCR	PAIRED	DE
SRR27235867	Fibroblast, normal cells from skin	-	NextSeq 550	Size fractionation	PAIRED	DE
SRR27235866	Fibroblast, normal cells from skin	-	NextSeq 550	Size fractionation	PAIRED	DE

*—The “Analysis” column indicates the type of analysis in which the data were used: Venn—primary comparison and Venn diagram reconstruction, DE—differential expression analysis.

**Table 2:** A list of TEs RNAs that are specific for different groups of transcriptome datasets as illustrated in the Venn diagram in Figure 2a.

Cell Types	DNA Transposons	Retroelements *	Pseudogenes	Unknown
**Expressed in ESCs, tumor tissues, and cancer cell lines but not in fibroblasts**	Charlie2a, Charlie2b, Tigger17b, Tigger5b, X4bDNA, Merlin1HS, Ricksha, Arthur1B, Arthur2, BLACKJACK, DNA1_Mam, Eulor11, Looper, MER113A, MER121B, MER45R, MER63A, MER97a,	L1M2a_5end, L1M2b_5end, L1M3c_5end, L1M3d_5end, L1M3e_5end, L1M4a1_5end, L1MC4_5end, L1MC5a_3end, L1MD2_5end, L1ME3C_3end, L1MEb_5end, L1MEd_5end, L1MEg_5end, L1MEi_5end, L1P4b_5end, L1P4c_5end, L1P4d_5end, L4B, CR1_Mam, HAL1b, PlatL3, HERV16, HERV30, HERVK11D, HERVL40, HUERS-P2, ERV24_Prim, ERVL47, LTR10B2, LTR21C, LTR26B, LTR26C, LTR2752, LTR27E, LTR37B, LTR38-int, LTR39-int, LTR48B, LTR53B, LTR53-int, MamGypsy2-I, MLT1F-int, MLT1G, MLT1J1, MLT1J2, PABLB-int, LTR37-int, MER34-int, MER70-int, MER83A-int, MER84-int, MER92B, MER92-int, MER66-int, MamRep1151, MER110A, MER67D	SSU-rRNACel	REP522
**Expressed in tumor tissues and cancer cell lines**	Arthur1A, Charlie11, Charlie16, Charlie17, Charlie7, Charlie7a, EuthAT-2, EutTc1-N2, hAT-N1_Mam, Helitron1Nb_Mam, Kanga1b, Tigger12A, Tigger15a, Tigger16a, Tigger6, MER97b, MER97d, MER81, MER96, MER20B, MER46C, MER58C, MER112, MER106A, MamTip3, MER99, UCON33, UCON9, X1DNA, X11DNA, X26DNA, Zaphod, Zaphod3	CR1Amni-1, CR1-16AMi, L1M2a1_5end, L1M7_5end, L1M8_5end, L1MCc_5end, L1ME3F_3end, L1ME4c_3end, L1MEa_5end, L1PA17_5end, L2b_3end, L3, L4C, L5 ERV3-16A3I, HERV-Fc1, LTR3, HERVL32, LTR16, LTR16A, LTR16B1, LTR33B, LTR40A1, LTR47B, LTR50, LTR52-int, LTR55, LTR58, LTR68, LTR69, LTR85c, LTR87, LTR91, MER110, MER110-int, MER76-int, MER76, MER89-int, MLT1H2, MLT1H-int, MLT1J-int, MLT1L, PrimAX-int, MER70B, MER70C, MER74C, MER90, MER92A, MER45A, MamRep605b	tRNA-Ala-GCY tRNA-Ala-GCY tRNA-Gly-GGG tRNA-Leu-CTA tRNA-Thr-ACG tRNA-Val-GTA	MER125 UCON20 UCON21 UCON23 UCON28a UCON64 UCON68
**Expressed only in ESCs**	Tigger13a, Tigger8, Arthur1C, Charlie14a, EutTc1-N1, X6a_DNA	LTR33A, LTR82B, MLT2E, MER110A		
**Expressed only in fibroblasts**	MER94, MER102a, MER123	LTR75, LTR16A2		

*—LINEs are shown in red, LTR/ERV in blue.

**Table 3:** A list of TE RNAs that are specific for cancer cell lines and their corresponding tumors as illustrated in the Venn diagrams in Figure 3.

Cell Types	DNA Transposons	Retroelements	Pseudogenes	Unknown
LUAD tissue	Tigger9a, Charlie1, Tigger5b, Charlie2b, Charlie7a, Tigger15a Kanga1d UCON21 MER63C MER46C MER119, UCON9 Looper, MER97a, UCON33, UCON23 MER99 MER125	MER66-int, L1M3e_5end,, LTR16, L1MEa_5end, L1M3b_5end, L1MEg_5end, L1ME3C_3end, LTR16A, L1MEb_5end, L1MC5a_3end, LTR53, MER68B, L1M3de_5end, LTR67B, HERVL32, MER110, LTR87, LTR55, MER76, HERVK11D, MER74B, L1M4a2_5end, L1ME3Cz_3end, MER34,LTR47B3, HERV1_LTRe, L1ME5_3end,, MER131, MER73, LTR16E1, LTR1C3, LTR27D, HERV-Fc1_LTR3, LTR53-int, LTR39-int, LTR58, LTR26C, MER101B, LTR34, MER21-int, MLT1L, LTR40A1	tRNA-Tyr-TAT	UCON64, UCON28a
LUAD cell line H1975	Charlie18a Merlin1_HS MamTip3 MER45A MER104	MamRep1151 LTR65 L1M2a1_5end.LTR16B1 LTR68 MER92A L1P4c_5end MER41G HERV1_I L1ME3G_3end, LTR10B2 MER20B LTR69		
LUAD cell line A549	MER112	HERVL74, LTR43-int, MLT1G MLT1J2, AluSg	tRNA-Pro-CCA, tRNA-Pro-CCY, tRNA-Ala-GCG, tRNA-Thr-ACG, tRNA-Ala-GCY_v,	
NB tissue	Arthur1A, Tigger7, Charlie7, Arthur1B, MER121B, MER115, Ricksha_0	L1M3b_5end, L1MDb_5endб MER68B, LTR75_1, MER84-int, MLT1G, HERV15, MLT1E L1MEi_5end, L1P4c_5end, LTR39-int, L4_B_Mam, L1M3d_5end, MER21-int, LTR48B	tRNA-Pro-CCA, tRNA-Ala-GCY_v, tRNA-Leu-CTA, tRNA-Glu-GAG_v, tRNA-Leu-TTG	UCON68
NB cell line SK-N-SH	Charlie18a, Tigger15a, Tigger6b, Charlie4z, Kanga2_a, Kanga1c, hAT-N1_Mam, Tigger2b_Pri, Tigger12c, Charlie5, Tigger9b, MER44D, FordPrefect, MER46C, MER63B, MER96, UCON21, hAT-5_Mam, MER113A, MER81, Eulor11, MER58D, EutTc1-N2, MER63D, DNA1_Mam ORSL, MER47B, MER125, Tigger14a	LTR72,, L1PREC2_5end, MER65B, MER57E1, MER61C, MER57C2,MLT2B1, L1ME3D_3end, LTR38A1,LTR82A,, LTR41, MLT1E1, LTR35B, MER68-int, MER72B, MLT2B5, MLT-int, L1M2c_5end, HERVL74,LTR26B, L1ME3C_3end, MLT1G3,HERV1_LTRb, LTR16A, MLT1E2, LTR31, AmnSINE2, LTR40b, LTR1B0, MLT1E1A, LTR23-int, LTR53B, LTR38, MER67A, L1MEf_5end, LTR2752, MER66C, LTR33B, MER5C1, LTR29, LTR52, MER50C, MER66D, MER76, L1ME4a_3end, MER61B, LTR21C, LTR1C1, LTR18A, L1ME3Cz_3end, LTR24C, MER61D, MER74C, MER73, MER92B, LTR1C3, LTR27D, L1MCb_5end, L1MEc_5end, LTR27C, HERVIP10B3, HERVFH19, MER52-int, L1ME3G_3end, LTR34, LTR10B2, LTR10B, MER88, MER89, MamRep605, LTR1F2, LTR16C, MLT2F, MLT1F, L1P4a_5end, MER67B, MER77B, L2c_3end	tRNA-Pro-CCY, tRNA-Val-GTY, tRNA-Ser-TCG, tRNA-Thr-ACG, tRNA-Leu-TTA, tRNA-Tyr-TAT, tRNA-Gln-CAA, tRNA-Pro-CCG, tRNA-Met_v, tRNA-Phe-TTY, tRNA-Leu-CTYtRNA, -Ala-GCY	

**Table 4:** Cell-type-specific patterns of TEs expression in LUAD at the first level of single-cell reads clustering.

Cell Type	DNA Transposons	Retroelements	Pseudogenes	Unknown
**Epithelial**		LTR28C, LTR1D1, LTR26B, MER4CL34, HERVL66, LTR57, LTR41C, L1M2a_5end, HERVP71A, MER67A, LTR52, L1PA17_5end, HERV15, LTR21C, L1ME3Cz_3end, HERV-Fc1, MER92-int, LTR22, MER34C_v, HERVIP10B3, MER61F, MER77, L2, HERV3		REP522
**Endothe-** **lial**	X11_DNA, Tigger17d, Tigger5b, MER106AMER47B	MER51D MER61C L1M3b_5end L1MEb_5end MER34C LTR16A2, MER83C, MLT1-int		UCON64
**Immune**		MER101, LTR72, LTR26E, HERV9, LTR28, LTR47B2, LTR48		
**Stromal**	Charlie18a, Ricksha, MER45B, UCON23, MER103C	L1MC5_3end, L1ME3D_3end, LTR38A1, L1ME3, B_3end, MER67D, LTR28B, L1MEg_5end, LTR18C, HERVL32, LTR59, HERV-Fc1-LTR2, L2b_3end, LTR19C, LTR27C, MLT1I		

**Table 5:** Cell-type-specific patterns of TEs expression in LUAD at the second level of single-cell reads clustering.

Cell Type (Number of TEs)	DNA Transposons	Retroelements	Pseudogenes	Unknown
**LUAD airway EP(24)**		MER66-int, MER65B, LTR26B, MLT1G3, HERVL66, MER34A1, LTR52, LTR45, L1PA17_5end, HAL1M8, HERVK11D, HERV15, LTR21C, L1ME3Cz_3end, HERV4_I, MER73, LTR22, MER83B, MLT1F, LTR1E, HERV3, L2c_3end		REP522
**LUAD alveolar EP (6)**	MER105	LTR18A, MER92-int, MER88, MER89		
**Blood vessels (13)**	Tigger5b, Tigger17d, UCON9	MER51D, MER61C, L1MEb_5end, LTR31, LTR37B, LTR16A2, MER83C, MLT1-int		UCON64
**Fibroblast lineage (11)**	Ricksha, Charlie18a, MER103C, UCON23	L1ME3D_3end, L1ME3B_3end, L1MEg_5end, HERV-Fc1_LTR2, L2b_3end, LTR19C, MLT1I		
**Lymphatic cells (8)**	Ricksha_b, MER58D	L1MC5_3end, MLT1H, L1MD2_5end, LTR2752, HAL1, MER74B		
**Lymphoid cells (3)**		LTR72, MLT1E3, LTR40c		
**Mesothelium (5)**	Ricksha_a	MER66A, LTR1B0, LTR23-int, MER101-int		
**Myeloid (5)**	-	LTR47B4, LTR54B, MER54B, MLT1G, MER52A		
**Smooth muscle (6)**	Zaphod3, MER8	MLT1E1, L1M3c_5end, MLT1E, LTR39-int		

## Data Availability

All the data are presented in Supplementary Files.

## Supplementary Materials

The following supporting information can be downloaded at: https://www.mdpi.com/article/10.3390/cimb46080505/s1, Figure S1 (Volcano plots of differentially expressed TEs in multiple myeloma (MM) cells vs embryonic stem cells (ESC) or vs fibroblasts), Table S1 (Full list of TEs for Figure 2 and Figure 4a), Table S2 (TE differential expression data)), Supplementary Files S1–S8, quality control files (zipped as a single zip-file).

## Glossary

*Autonomous transposon*	a transposon that encodes its own enzymes for transposition
*BLACKJACK*, *Looper*, *Zaphod*, *X* and *Tigger*	ancient DNA transposons. Some X elements are non-autonomous retroelements.
*DNA transposons*	Class II mobile elements that transpose directly from one site to another using a “cut-and-paste” mechanism. DNA transposons have no reverse transcriptase domains and usually have terminal inverted repeats, flanking core-sequence-encoding transposase.
*Embyonic stem cells (ESCs)*	cells of the inner cell mass of the blastocyst, an early stage of the developing embryo that lasts from 4 to 7 days after fertilization.
*Endogenous retroviruses (ERVSs)*	inherited genetic elements derived from exogenous retroviral infections occurring throughout the evolution.
*Eulor (euteleostomi-conserved low-frequency repeat)*	a family of unclassified ancient repeats.
*EUTREP* (eutherian repeat)	a family of ancient repeats that is not attributed to any class of DNA repeats.
*HERVs—human endogenous retroviruses*	a group of viral elements present in the human genome that bear resemblance to contemporary exogenous retroviruses.
*LINEs, long interspersed nuclear elements*	autonomous non-LTR retrotransposons
*Long non-coding RNA (lncRNA)*	transcript that does not encode a protein and is longer than 200 base pairs. lncRNAs are (Pol I)-, Pol II-, and Pol III-transcribed RNAs, as well as RNAs from processed introns
*LTRs—long terminal repeats*	direct simple repeats flanking the core sequence of some types of retrotransposons.
*MER*	medium reiterated frequency repeats—transposons of various families in human genome.
*MLT*	mammalian LTR transposon—a family of ERVs specific to mammals
*Non-LTR retrotransposons*	retrotransposons that lack long terminal repeats.
*Pseudogene*	a DNA segment that has a structural resemblance to a gene; however, it lacks the ability to encode a protein.
*Retrotransposons*	Class I mobile elements that have reverse transcriptase domains. These elements use reverse transcription and replicate themselves in the genome during the transposition process.
*SINEs, short interspersed nuclear elements*	non-autonomous, non-LTR retrotransposons that utilize the enzymatic machinery of LINEs for transposition.
*The tumor microenvironment (TME)*	refers to the dynamic structure surrounding cancer cells and interacting with them. TME includes immune cells, the extracellular matrix, blood vessels, and other cell types, such as fibroblasts.
*Transcripts per million (TPM)*	is a normalization method for RNA-seq. The abbreviation means that “for every 1,000,000 RNA molecules in the RNA-seq sample, *TPM value* came from this gene/transcript.”
*Transposable or mobile element (TE)*	a sequence of DNA that is capable of changing its location in the genome.
*Trimming*	a process that precedes the assembly of a genome or its analysis. Trimming is a crucial preliminary step in genome assembly or analysis. This process involves the removal of low-quality bases, identified by their high likelihood of incorrect calling, as well as the elimination of adapter sequences
*UCON* (ultraconserved element)	a family of ancient repeats that are not classified yet
