# Supplementary material for: Dysregulation of Transposon Transcription Profiles in Cancer Cells Resembles That of Embryonic Stem Cells
Source: Curr Issues Mol Biol. 2024 Aug 5;46(8):8576–99. doi: 10.3390/cimb46080505 (PMC11353194; doi:10.3390/cimb46080505)
Supplement: Supplementary file 1 [file cimb-46-00505-s001.zip › Supplementary Figure S1.pdf]

current issues in molecular biology

MDPI

Supplementary Materials

Dysregulation of Transposon Transcription Profiles in Cancer Cells Resembles That of Embryonic Stem Cells

Anna I. Solovyeva [1,2], Roman V. Afanasev [1], Marina A. Popova [1,3] and Natella I. Enukashvily [1,4,*]

1 Lab of the Non-Coding DNA Studies, Institute of Cytology, Russian Academy of Sciences, 194064 St. Petersburg, Russia
2 Zoological Institute of Russian Academy of Sciences, 199034 St. Petersburg, Russia
3 Applied Genomics Laboratory, SCAMT Institute, ITMO University, 191002 St. Petersburg, Russia
4 Department of Cytology and Histology, St. Petersburg State University, 199034 St. Petersburg, Russia
* Correspondence: n.enukashvily@incras.ru

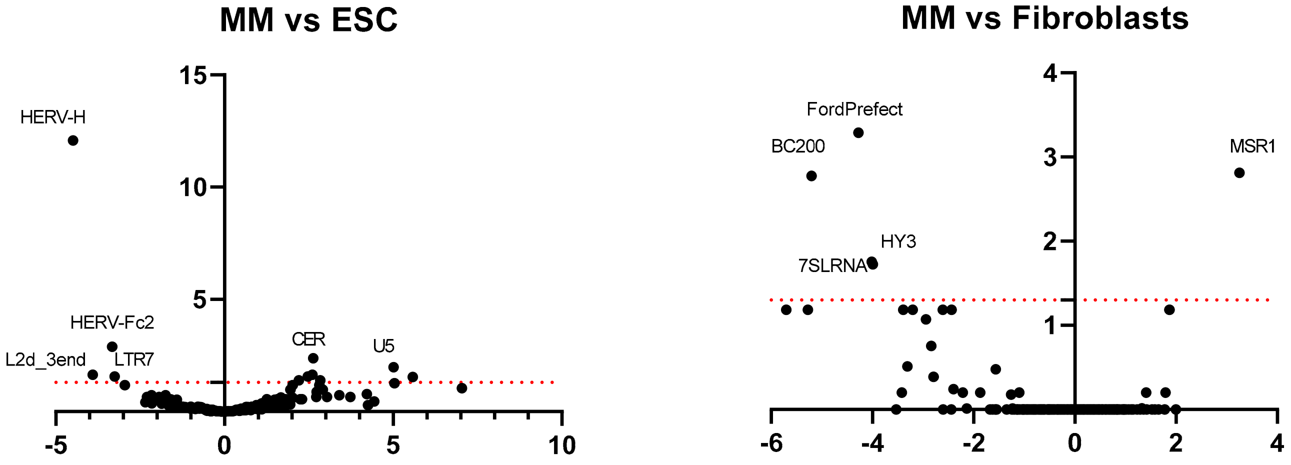

Supplementary Figure S1. Volcano plots of differentially expressed TEs in multiple myeloma (MM) cells vs embryonic stem cells (ESC) (*left column*) or vs fibroblasts (*right column*),. *X*-axis—*b* or *beta*-value (log_2 fold changes between conditions) calculated by the Sleuth package. *Y*-axis—−log(q-value); the red dotted line corresponds to q-value < 0.05, the dots above the line are either up-regulated (b < 0) or downregulated (b > 0).
